# Designing biochars for improved sorptive removal of per‐ and polyfluoroalkyl substances

**DOI:** 10.1002/jeq2.70195

**Published:** 2026-05-13

**Authors:** Wei Zheng, Erin Huggett, Sophie Circenis, Kalidas Mainali, Brajendra Sharma

**Affiliations:** ^1^ Illinois Sustainable Technology Center University of Illinois at Urbana‐Champaign Champaign Illinois USA; ^2^ USDA‐ARS Eastern Regional Research Center, Sustainable Biofuels and Co‐Products Research Unit Wyndmoor Pennsylvania USA

## Abstract

The widespread occurrence of per‐ and polyfluoroalkyl substances (PFAS) in aquatic environments, along with their adverse impacts on human health, has been recognized as an emerging issue. Sorption using carbon‐based sorbents is the most common approach for PFAS removal. However, conventional biochars typically underperform compared to activated carbon, which is effective but relatively costly. In this study, a designer biochar was developed by using lime sludge, a no‐cost byproduct from drinking water treatment plants, as a pretreatment for woody biomass followed by systematic optimization of lime‐sludge loading, pyrolysis temperature, and residence time. The results showed that the pretreatment process enhanced pore development and modified the surface chemistry of the designer biochar. Under optimal conditions (1:4 lime sludge‐to‐biomass, 850°C, 5 h), the designer biochar achieved near‐quantitative removal (>99%) of perfluorooctanoic acid and both linear and branched perfluorooctane sulfonate, outperforming unmodified biochar and comparable to a commercial activated carbon. Sorption kinetics and isothermal studies confirmed the superior performance of the designer biochar, exhibiting rapid equilibration (<1 h) and increased sorption capacity. Mechanistic analysis revealed that improved PFAS removal by the designer biochar was driven by multiple adsorption mechanisms, including hydrophobic interactions, electrostatic attraction, and cation bridging facilitated by Ca^2^^+^ and Mg^2^^+^ species derived from lime sludge. This study highlights lime sludge pretreatment as a sustainable and cost‐effective strategy for producing high‐performance carbon‐based sorbents for PFAS remediation in contaminated water systems.

## Core Ideas

- Lime sludge pretreatment enhances pore development and modifies biochar surface chemistry.
- Pretreatment significantly improves per‐ and polyfluoroalkyl substances (PFAS) sorption by designer biochar.
- Designer biochar exhibits rapid sorption kinetics and high sorption capacity.
- Optimized designer biochar achieves >99% removal of perfluorooctanoic acid (PFOA) and perfluorooctane sulfonate (PFOS).
- Synergistic mechanisms drive enhanced PFAS sorption in designer biochar.

## Plain Language Summary

Per‐ and polyfluoroalkyl substances (PFAS), often called “forever chemicals,” are human‐made compounds that resist breakdown and have been detected in water, wildlife, and people around the world. Because of their persistence and potential health risks, finding effective ways to remove these contaminants from water is an emerging challenge. In this study, we developed a designer biochar by treating woody biomass with lime sludge, a byproduct of drinking water treatment. This process enhanced the biochar's structure and surface properties, allowing it to remove PFAS as effectively as, or even better than, costly commercial activated carbon. The designer biochar acted rapidly and captured PFAS through multiple interactions. These findings demonstrate that lime sludge pretreatment offers a sustainable, low‐cost method to produce high‐performance carbon‐based sorbents for cleaning PFAS‐contaminated water.

**Abbreviations**
AC: activated carbon
GAC: granular activated carbon
PFAS: per‐ and polyfluoroalkyl substances
PFOA: perfluorooctanoic acid
PFOS: perfluorooctane sulfonate
SSA: specific surface area
UPLC‐MS/MS: ultra‐performance liquid chromatography coupled with tandem mass spectrometry

## 1 INTRODUCTION

Per‐ and polyfluoroalkyl substances (PFAS) are a large class of anthropogenic chemicals extensively used in everyday products, including nonstick cookware, grease‐resistant textiles, food wrapping materials, and fire‐fighting foams. Owing to their remarkable thermal, chemical, and biological stability, PFAS are often referred to as “forever chemicals.” Their extensive use and exceptional persistence have resulted in widespread occurrence across environmental media and within wildlife (Giesy & Kannan, 2001; Nakayama et al., 2019; Remucal, 2019). Over the past two decades, numerous PFAS, particularly perfluoroalkyl acids such as perfluorooctane sulfonate (PFOS) and perfluorooctanoic acid (PFOA), have been frequently detected in drinking water sources (Giesy & Kannan, 2001; Nakayama et al., 2019; Remucal, 2019; Tokranov et al., 2024). In the United States, PFOS and PFOA concentrations in drinking water have been reported at multiple sites, ranging from a few nanograms per liter to several tens of nanograms per liter (Boone et al., 2019; Crone et al., 2019; Lindstrom et al., 2011; Tokranov et al., 2024). The widespread occurrence of PFAS in water systems, coupled with growing evidence of their adverse effects on human health (Domingo & Nadal, 2019; Li et al., 2025), has emerged as a significant environmental concern (Wilder et al., 2017).

Physical separation treatments such as activated carbon (AC) sorption (McCleaf et al., 2017; Xiao et al., 2017), membrane filtration (Johnson et al., 2019), and resin ion exchange (Huang et al., 2018; McCleaf et al., 2017) are the most common and practical technologies to remove low‐concentration PFAS from contaminated water and wastewater. Commercially available granular activated carbon (GAC), the most widely used sorbent, has been applied in ex situ treatment systems for the removal of PFAS from contaminated water (McGregor, 2018; Ross et al., 2018). Biochar, a carbon‐rich sorbent, has recently emerged as a promising alternative to AC for PFAS removal from aqueous systems (Ao et al., 2024; Krebsbach, He, Adhikari, et al., 2023; Krebsbach, He, Oh, et al., 2023; Nasrollahpour et al., 2025; Petrangeli Papini et al., 2024; Xiao et al., 2017; Zhang et al., 2019). Typically produced from forestry and agricultural residues, biochar offers a cost advantage over AC (e.g., $200–1000 tonne^−1^ biochar vs. $1500–3000 tonne^−1^ GAC) (Zhang et al., 2019). Moreover, biochar production requires lower energy inputs and can contribute to carbon sequestration, supporting its potential as a more sustainable and environmentally friendly sorbent (Afrooz et al., 2025). However, unmodified (pristine) biochars generally exhibit lower PFAS sorption capacities than commercial AC (Niaz et al., 2024; Xiao et al., 2017), underscoring the need for further modification to enhance their sorption capacity.

To improve the PFAS removal efficiency of biochar, various thermal and chemical modification techniques have been explored (Afrooz et al., 2025; Nasrollahpour et al., 2025). These methods are conceptually similar to the activation processes used for producing AC from fossil fuels or coconut shells (Rehman et al., 2019). Thermal activation can increase biochar's surface area, pore volume, and pore size distribution (Krebsbach, He, Adhikari, et al., 2023), thereby improving its sorption capacity for PFAS (Ao et al., 2024; Z. Y. Wang, Alinezhad, Nason, et al., 2023; Z. Y. Wang, Alinezhad, Sun, et al., 2023). Such structural enhancements strengthen hydrophobic interaction between PFAS and the activated biochars. In addition to thermal activation, chemical modifications, including acid/base washing, polymer functionalization with positively charged groups, or incorporation of metal‐rich materials, have also been explored (Deng et al., 2023; Niaz et al., 2024; Z. Y. Wang, Alinezhad, Nason, et al., 2023; Yu et al., 2023). Acid or base treatment can remove impurities from carbonaceous sorbents, increase surface area and porosity, and introduce functional group (Rehman et al., 2019; Zhao et al., 2017), thereby creating more active sites for PFAS sorption. Incorporating polymer or iron oxides into biochar has also proven effective by promoting hydrophobic or electrostatic interactions with anionic PFAS (Deng et al., 2023; Z. Y. Liu et al., 2023; Niaz et al., 2024; Rodrigo et al., 2022). Moreover, doping biochars with metal cations could substantially reduce their negative surface charge (Rodrigo et al., 2022), weakening electrostatic repulsion and facilitating the sorption of anionic PFAS onto the modified biochars.

Pretreating biomass with metal‐rich materials prior to pyrolysis is another effective strategy for enhancing biochar's sorption capacity (Afrooz et al., 2025). Our previous studies demonstrated that biomass pretreated with calcium‐rich materials synergistically improved the capture of anionic species (e.g., phosphate) by designer biochars (Katuwal et al., 2023; Yang et al., 2021). Similarly, biochars synthesized from biomass pretreated with metallic oxides (e.g., MgO and Fe‐based nanoparticles) have exhibited enhanced PFAS sorption compared to their pristine biochars (Niaz et al., 2024). For example, Z. Y. Liu et al. (2023) synthesized magnetic biochars by pretreating maize straw with FeCl_3_ followed by ball milling, resulting in graphite‐like biochars with superior sorption capacities for both long‐ and short‐chain PFAS compared to commercial GAC and powdered AC. Despite these advances, most prior studies have relied on metal additives derived from potentially hazardous industrial byproducts. In contrast, lime sludge, a byproduct of drinking water treatment, is a safer and more readily available alternative to iron‐based or other metallic additives (e.g., ZnCl_2_) (Afrooz et al., 2025). However, the use of abundant and nonhazardous lime sludge as a biomass pretreatment agent to tailor biochar properties for PFAS removal remains underexplored.

In this study, we aim to design and evaluate a novel biochar derived from woody biomass pretreated with lime sludge. The objectives of the research are to (1) optimize production conditions for generating high‐performance designer biochar; (2) systematically investigate the sorption kinetics and isotherms of PFOS and PFOA on the designer biochar; and (3) elucidate the improved PFAS sorption mechanisms compared to the unmodified biochar. The outcomes of this work will provide critical insights for developing designer biochars with tailored physicochemical properties for effective PFAS remediation from contaminated water.

**Core Ideas**

- Lime sludge pretreatment enhances pore development and modifies biochar surface chemistry.
- Pretreatment significantly improves per‐ and polyfluoroalkyl substances (PFAS) sorption by designer biochar.
- Designer biochar exhibits rapid sorption kinetics and high sorption capacity.
- Optimized designer biochar achieves >99% removal of perfluorooctanoic acid (PFOA) and perfluorooctane sulfonate (PFOS).
- Synergistic mechanisms drive enhanced PFAS sorption in designer biochar.

## 2 MATERIALS AND METHODS

### 2.1 Chemicals and materials

PFOS (potassium salt, ≥98.0%) and PFOA (>96.0%) were purchased from Sigma‐Aldrich. Stock solutions of PFOS and PFOA were prepared in methanol, and sorption solutions were made by diluting each PFAS stock in nanopure deionized water (>18.0 MΩ‐cm). The methanol content in the sorption solutions was maintained below 0.1% to minimize potential solvent effects on PFAS sorption by carbon‐based sorbents. Isotopically labeled standards, perfluoro‐n‐[1,2,3,4‐13C_4_]octanoic acid (^13^C_4_‐PFOA) and perfluoro‐n‐[1,2,3,4‐13C_4_]octanesulfonate (^13^C_4_‐PFOS), were obtained from Wellington Laboratories. Other analytical‐grade chemicals, including methanol, were purchased from Thermo Fisher Scientific.

Woody biomass, specifically sawdust from pine tree (*Pinus* spp.), was collected from the University of Illinois Energy Farm in Urbana, IL. The material was air‐dried and sieved to obtain particles ˂1.0 mm. Lime sludge, a byproduct of the water‐softening process and primarily composed of CaCO_3_ with high magnesium content (Yang et al., 2021), was collected from a drinking water treatment facility in Champaign, IL. A commercial AC (Darco, G‐60) was purchased from Aldrich Chemical Company.

### 2.2 Designer biochar production

A laboratory‐scale pyrolysis system was used to produce designer biochars following methods described in our previous studies (Katuwal et al., 2023; Yang et al., 2021). Briefly, lime sludge slurry was thoroughly mixed with biomass at predetermined ratios. The feedstock mixture (200 g) was weighed and loaded into the pyrolysis reactor. Pyrolysis was conducted by heating the feedstock to the target temperature at a rate of 10°C·min^−^^1^ under a continuous N_2_ flow of 2 L·min^−^^1^, followed by holding at the target temperature for a specified duration. After pyrolysis, the biochars were allowed to cool to ambient temperature within the reactor before being transferred to airtight containers.

To generate the most efficient and cost‐effective designer biochars, four production experiments were conducted to evaluate critical processing parameters:

1. *Effect of lime sludge‐to‐biomass ratio on PFAS sorption*: Lime sludge was mixed with sawdust at ratios of 0, 1:20, 1:4, and 1:1 (lime sludge/biomass, dry weight basis). The mixtures were pyrolyzed at 850°C for 2 h.
2. *Effect of pyrolysis temperatures on PFAS sorption*: A 1:4 (lime sludge/sawdust, dry weight basis) mixture was pyrolyzed at target temperatures of 650°C, 850°C, 900°C, and 1000°C (heating rate: 10°C min^−^^1^), with a holding time of 5 h. Unmodified biochars produced from biomass without lime sludge under identical conditions were used for comparison.
3. *Effects of pyrolysis duration on PFAS sorption*: Sawdust mixed with lime sludge at ratios of 1:20 and 1:5 (dry weight basis) was pyrolyzed at 850°C for either 2 or 5 h. Pine sawdust without lime sludge addition was also pyrolyzed under the same conditions to produce unmodified biochars.
4. *Effects of feedstock pretreatment and biochar post‐modification on PFAS removal*: Unmodified biochar, designer biochar, and lime sludge were produced via pyrolysis at 850°C for 5 h. Designer biochar was produced from sawdust pretreated with lime sludge at a 1:4 ratio (lime sludge/biomass, dry weight basis). Post‐modified sorbents were prepared by physically mixing unmodified biochar with either lime sludge or pyrolyzed lime sludge at a 1:1 (dry weight basis) ratio.

### 2.3 Sorption experiments

A series of batch experiments was conducted to evaluate the PFAS sorption capacities of all produced biochars. In brief, 0.01 g of each biochar sample was weighed into a glass Erlenmeyer vessel, followed by the addition of 50 mL of an aqueous solution containing 0.25 mg L^−^^1^ each of PFOS and PFOA. The sealed vessels were agitated on a reciprocal shaker at 190 rpm and 21.0 ± 0.5°C. After 24 h of shaking, the suspensions were centrifuged at 14,000 rpm for 10 min. Supernatants were transferred to 1.0‐mL volumetric flasks and spiked with 0.01 mL of a 200 ng mL^−^^1^ isotopically labeled internal standard solution. PFAS concentrations in the supernatants were quantified using ultra‐performance liquid chromatography coupled with tandem mass spectrometry (UPLC‐MS/MS). Adsorbed amounts were calculated by subtracting the final concentrations from the initial PFAS concentrations. Blank experiments without sorbents were performed concurrently and showed no significant PFAS loss during the sorption process. All experiments were carried out in triplicates, and error bars represent the standard deviations of triplicate determinations. Differences in sorption capacities among biochars were analyzed using multivariate analysis of variance with a significance level of *p* < 0.05.

Sorption kinetics and isotherm experiments were conducted to further assess the PFAS removal performance of the designer biochar produced under optimal pyrolysis conditions. For the kinetics study, 0.01 g of sorbent was added to 50 mL of a 0.25 mg L^−^^1^ PFOA or PFOS solution and shaken at 190 rpm for contact times ranging from 5 to 1440 min. For isotherm experiments, 0.01 g of sorbent was mixed with 50 mL of PFAS solutions with a concentration gradient of 0.1–5 mg L^−^^1^ for PFOA and 0.25–10 mg L^−^^1^ for PFOS. Samples were shaken at 190 rpm for 24 h to ensure equilibrium, then centrifuged, and the supernatants analyzed to determine PFAS sorption onto the sorbents. The coefficient of determination (*R* ^2^), standard deviation, and other statistics were analyzed by OriginPro 2024b.

### 2.4 PFAS analysis and biochar characterization

Concentrations of PFAS in the supernatants were determined using a Shimadzu 8050 UPLC‐MS/MS according to the US EPA method 1633 with some modifications. The targeted PFAS compounds were separated on a Waters Acquity C_18_ column (1.7‐µm particle size, 2.1 mm × 100 mm; Waters) by a UPLC system (LC‐40D, Shimadzu). A gradient elution was employed using two mobile phases: solvent A (water with 0.1% ammonium acetate) and solvent B (5:95 water:acetonitrile). The gradient program started with 80% solvent A and 20% solvent B, maintained for 0.1 min, then linearly ramped to 10% solvent A and 90% solvent B over 9.0 min and held for 3.0 min. The gradient then returned to the initial conditions. The flow rate was set at 0.3 mL min^−1^. A triple quadrupole mass spectrometer (LCMS‐8050, Shimadzu) equipped with an electrospray ionization source operated in negative mode was used for quantification. Optimized MS conditions included a nebulizing gas flow of 3 L min^−1^, heating gas flow of 15 L min^−1^, and drying gas flow of 5 L min^−1^. Quantitative analysis was performed in multiple reaction‐monitoring modes. Optimized MS parameters, retention time, instrumental limits of detection, and limits of quantification for the targeted PFAS compounds are provided in Table S1.

The elemental composition of all biochar samples was analyzed using a CHNS/O elemental analyzer (Flash EA 1112, Thermo Scientific). Nitrogen (N_2_) adsorption–desorption isotherms were measured at 77 K using an automated adsorption analyzer (Autosorb iQ with ASiQwin software, Quantachrome). Prior to measurement, all samples were degassed at 385°C under vacuum for 3 h. Specific surface areas (SSAs) and pore volumes were determined from the N_2_ adsorption–desorption isotherms using the Brunauer–Emmett–Teller method and density functional theory model, respectively. Additional physicochemical properties, including ash content and pH, were characterized following methods described in our previous studies (Katuwal et al., 2023; Yang et al., 2021). The zeta potentials of the unmodified and designer biochars across different pH values were measured using a Litesizer 500 particle analyzer (Anton Paar).

## 3 RESULTS AND DISCUSSION

### 3.1 Effects of additive ratios with biomass on PFAS sorption capacities of designer biochars

**FIGURE 1:**
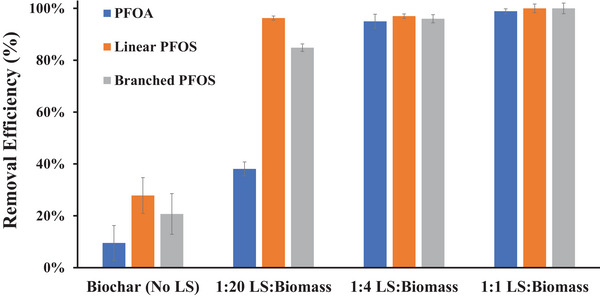
Effect of lime sludge (LS) addition amounts on designer biochar sorption of perfluorooctanoic acid (PFOA) and perfluorooctane sulfonate (PFOS).

A series of designer biochars were synthesized by pyrolyzing sawdust biomass pretreated with varying amounts of lime sludge at 850°C for 2 h. Compared to biochar produced from biomass alone, the designer biochars exhibited substantially enhanced removal efficiencies for PFOA and both linear and branched isomers of PFOS (Figure 1). The sorption capacities of all designer biochars were significantly higher than those of the unmodified biochar (*p* < 0.05; Table S2), indicating that lime sludge pretreatment significantly improves the PFAS sorption performance of biochar.

For both the unmodified biochar and the designer biochar with low lime sludge content (i.e., 1:20 sludge‐to‐biomass ratio), linear PFOS exhibited the highest removal, followed by branched PFOS and PFOA. This observation is consistent with previous observations that linear PFOS exhibits stronger sorption potential than its branched isomers, primarily due to differences in molecular configuration and polarity (Schulz et al., 2020). It also aligns with earlier studies on carbon‐based sorbents, such as biochar and AC, which have demonstrated greater sorption affinity for PFAS containing sulfonate functional groups compared to those bearing carboxylate groups (Hansen et al., 2010; N. Liu et al., 2021; Niaz et al., 2024). At higher lime sludge‐to‐biomass ratios (1:4 and 1:1), removal efficiencies exceeded 95% for all three PFAS species for both designer biochars (Figure 1). Sorption capacities for PFOA and branched PFOS were significantly greater than those observed for the designer biochar produced at a 1:20 ratio (Table S2), indicating that increased lime sludge loading enhanced sorption site availability. Based on these results, a lime sludge‐to‐biomass ratio of 1:4 was selected for subsequent designer biochar production.

### 3.2 Effects of pyrolysis temperatures and duration on PFAS sorption capacities of designer biochar

**FIGURE 2:**
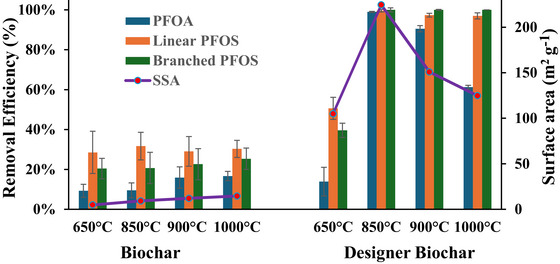
Effect of pyrolysis temperature on perfluorooctanoic acid (PFOA) and perfluorooctane sulfonate (PFOS) removal by biochar and designer biochar. Specific surface area (SSA) is special surface area (m^2^ g^−^^1^).

In general, the physicochemical properties and sorption capacities of biochars are primarily influenced by two key production parameters: pyrolysis temperature and duration (N. Liu et al., 2021). Figure 2 illustrates the effect of pyrolysis temperature on the removal rates of PFOA and PFOS by unmodified biochars and designer biochars from PFAS‐containing aqueous solutions. For the unmodified biochars, increasing the pyrolysis temperature led to a modest improvement in PFAS removal efficiency (Figure 2). As the temperature increased from 650°C to 1000°C, the removal rates of PFOA, linear PFOS, and branched PFOS rose from 9.3% to 16.7%, 28.5% to 30.3%, and 20.4% to 25.3%, respectively. This moderate improvement is consistent with the corresponding increase in the SSA of the unmodified biochars, which rose from 4.79 to 14.5 m^2^ g^−1^ over the same temperature range (Figure 2; Table S3).

In contrast, designer biochars exhibited a dramatic increase in PFAS removal efficiency at a pyrolysis temperature of 850°C (Figure 2). At this temperature, removal rates exceeded 99% for all three PFAS compounds. However, further increasing the temperature beyond 850°C resulted in a slight decline in PFOA removal, coinciding with a reduction in SSA from 224.5 m^2^ g^−^^1^ at 850°C to approximately 124.7 m^2^ g^−^^1^ at 1000°C (Table S3). The total pore volume also decreased from 0.122 to 0.079 cm^3^ g^−^^1^ over the same temperature range. The reduction in SSA and pore volume at elevated temperatures may be attributed to pore collapse and/or ash fusion (Ao et al., 2024), which can occur during high‐temperature pyrolysis and limit the development or accessibility of sorption sites. Considering both energy input and adsorption efficiency, 850°C was identified as the optimal pyrolysis temperature for producing designer biochars.

Pyrolysis duration also plays a critical role in governing PFAS sorption by biochars (N. Liu et al., 2021). Figure S1 illustrates the effect of pyrolysis duration on PFOA and PFOS removal by both unmodified and designer biochars at 850°C. Although biomass carbonization is typically completed within 30 min under these conditions, achieving optimal PFAS sorption required a minimum pyrolysis duration of 2 h. Extending the duration to 5 h resulted in the highest removal efficiencies for all PFAS compounds (Figure S1). A previous study indicated that prolonged pyrolysis may enhance carbon structure and improve surface hydrophobicity (N. Liu et al., 2021). This study demonstrated that increasing the pyrolysis duration from 2 to 5 h increased the SSA and total pore volume of the designer biochar from 169.3 m^2^ g^−^^1^ and 0.080 cm^3^ g^−^^1^ to 224.5 m^2^ g^−^^1^ and 0.122 cm^3^ g^−^^1^, respectively (Table S3). The increase in surface area and pore volume facilitated stronger hydrophobic interactions between PFAS and the designer biochar, thereby enhancing its removal performance. Accordingly, the designer biochar derived from sawdust pretreated with lime sludge (at a 1:4 lime sludge‐to‐biomass ratio) and pyrolyzed at 850°C for 5 h was selected as the optimized sorbent for subsequent studies.

### 3.3 Pretreatment versus post‐modification for PFAS removal

**FIGURE 3:**
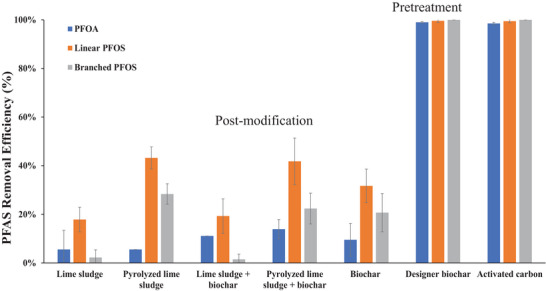
Comparative analysis of perfluorooctanoic acid (PFOA) and perfluorooctane sulfonate (PFOS) removal by unmodified biochar and designer biochar with pretreatment and post‐modification. Error bars indicate standard deviations from replicate experiments. PFAS, per‐ and polyfluoroalkyl substances.

Figure 3 compares the PFAS removal efficiencies of various sorbents, including the optimized designer biochar, lime sludge, pyrolyzed lime sludge, raw biochar, and commercial AC. The designer biochar, raw biochar, and pyrolyzed lime sludge were produced under the identical pyrolysis conditions (i.e., 850°C for 5 h). The results show that unmodified biochar, lime sludge, and pyrolyzed lime sludge exhibited relatively low PFAS sorption capacities, particularly when compared to commercial AC (Figure 3). Furthermore, the direct addition of lime sludge or pyrolyzed lime sludge to biochar, representing post‐modification approaches, did not significantly improve PFAS removal efficiency. As illustrated in Figure 3, the removal rates for these mixtures were comparable to those of their individual components. This suggests that simple post‐modification (i.e., physical mixing of lime sludge with biochar) is ineffective, likely because it does not activate the lime sludge or substantially modify the surface characteristics of the biochar.

In contrast, pretreating biomass with lime sludge prior to pyrolysis to produce designer biochar led to complete removal of all three PFAS compounds: PFOA, linear PFOS, and branched PFOS (Figure 3). The performance of designer biochar was comparable to that of the commercial AC, demonstrating its efficacy as a sorbent for long‐chain PFAS. Compared to the post‐modification method, this pretreatment approach significantly enhances sorption performance for biochars. The improved PFAS removal by the designer biochar could be attributed to chemical and physical interactions between the biomass and lime sludge during pyrolysis, which promote higher SSA, larger pore volume, and modified surface chemistry. Overall, lime sludge pretreatment offers a simple yet highly effective strategy for producing high‐performance carbon‐based sorbents for PFAS removal.

### 3.4 Sorption kinetics of PFAS by biochar and designer biochar

The sorption kinetics of PFOA, linear PFOS, and branched PFOS by unmodified biochar and designer biochar are displayed in Figure S2. The results clearly demonstrate that the designer biochar exhibits a higher sorption capacity and reaches equilibrium more rapidly than the unmodified biochar (Figure S2). For example, rapid sorption kinetics were observed for both PFOS isomers on the designer biochar with equilibrium achieved within 0.5 h, whereas approximately 5 h were required for the unmodified biochar to reach equilibrium. The accelerated sorption kinetics of the designer biochar can be attributed to differences in adsorption mechanisms and its surface characteristics. Furthermore, previous studies have reported that carbon‐based sorbents, including AC, often require several days to reach sorption equilibrium due to the slow diffusion of PFAS molecules into intraparticle pores (Ao et al., 2024; Du et al., 2015; Inyang & Dickenson, 2017). In contrast, the rapid kinetics of the designer biochar indicate its potential as a more efficient and practical sorbent for PFAS removal under real‐world conditions with limited contact times.

Three commonly used kinetic models, that is, the pseudo‐first‐order, pseudo‐second‐order, and Elovich models, were applied to simulate the sorption processes of the targeted PFAS on both unmodified and designer biochars. The detailed model equations are provided in Supporting Information Text S1, and the best‐fit kinetic parameters are summarized in Tables S4–S6. In general, the pseudo‐first‐order and pseudo‐second‐order models describe mononuclear and binuclear sorption processes, respectively. The Elovich model is an empirical model that accounts for surface heterogeneity and potential desorption effects. Except for the pseudo‐first‐order model applied to PFOA sorption, all three models provided excellent fits to the experimental data (*R* ^2^ ≥ 0.936). The equilibrium sorption capacities (*Q_e_*) calculated from both the pseudo‐first‐order and pseudo‐second‐order models were consistently higher for the designer biochar than for the unmodified biochar across all PFAS species, indicating superior sorption performance. In addition, the sorption rate constants (*k*_1_, *k*_2_, and *α*) of the designer biochar were significantly higher than those of the unmodified biochar (Tables S4–S6), further confirming the former requires much less time to obtain sorption equilibrium. Overall, the kinetic analysis demonstrates that lime sludge‐incorporated designer biochar is a more efficient sorbent for PFAS removal than unmodified biochar.

### 3.5 Sorption isotherms of PFAS by biochar and designer biochar

**FIGURE 4:**
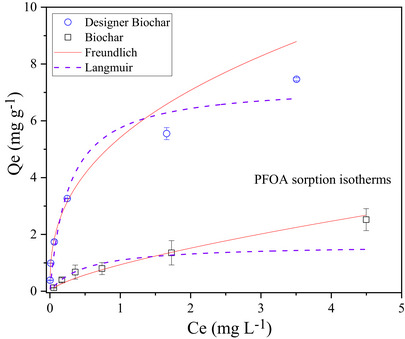
Sorption isotherms of perfluorooctanoic acid (PFOA) on biochar and designer biochar, fitted using Freundlich and Langmuir equations.

**FIGURE 5:**
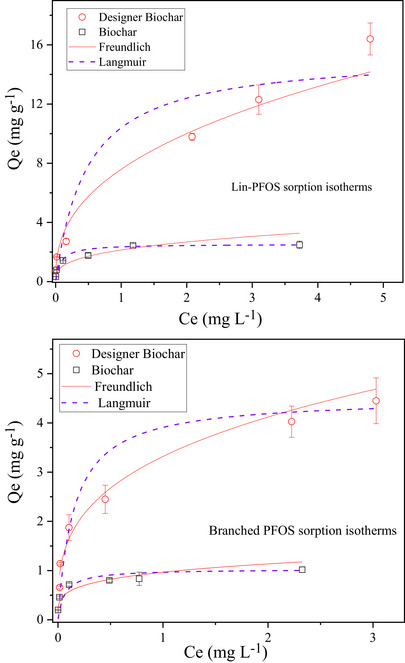
Sorption isotherms of linear and branched perfluorooctane sulfonate (PFOS) on biochar and designer biochar, fitted using Freundlich and Langmuir equations.

Figures 4 and 5 illustrate the sorption isotherms of PFOA, linear PFOS, and branched PFOS on the biochar and the designer biochar, respectively. Compared to unmodified biochar, the sorption isotherms for lime sludge‐incorporated designer biochar to PFAS are nonlinear with a concave‐down increasing trend (Figures 4 and 5), suggesting decreased sorption efficiency with increasing PFAS concentrations. The initial concentrations of PFOA and PFOS used in the isothermal sorption experiments were considerably lower than their respective critical micelle concentrations (Čabala et al., 2017; Y. Wang et al., 2015). Under these conditions, PFAS molecules likely adsorbed horizontally on the biochar surface, occupying only a portion of the available sorption sites (Yu et al., 2023); thus, the formation of hemimicelles or micelles during sorption was unlikely (Z. Y. Liu et al., 2023).

Two typical nonlinear equations, Langmuir and Freundlich (Supporting Information Text S1), were applied to simulate experimental sorption data (Figures 4 and 5). The correlation coefficients and the model constants for the three sorption isotherms are summarized in Table 1. Both models described the sorption behavior well for designer biochar (*R* ^2^ ≥ 0.922) and unmodified biochar (*R* ^2^ ≥ 0.928), suggesting that the interaction between PFAS and these biochars could be affected by both the Langmuir and Freundlich processes (Yao et al., 2013). This observation aligns with previous findings that PFAS sorption onto carbonaceous sorbents involves multiple mechanisms (Rodrigo et al., 2022; Yu et al., 2023). The maximum sorption capacities for all targeted PFAS calculated from Langmuir model are significantly higher in designer biochar than in biochar (Table 1). This study further confirms that designer biochar is a more effective sorbent than unmodified biochar for capturing PFAS.

**TABLE 1:** Sorption isotherm parameters for perfluorooctanoic acid (PFOA) and perfluorooctane sulfonate (PFOS) on biochar and designer biochar.

		Langmuir			Freundlich		
		*Q*_max_ (mg g^−1^)	*K_L_* (L mg^−1^)	*R* ^2^	*K_f_* (mg^(1 −^ *^n^* ^)^ L*^n^* g^−1^)	1/*n*	*R* ^2^
Biochar	PFOA	1.63 ± 0.20	2.02 ± 0.27	0.934	1.02 ± 0.08	0.65 ± 0.03	0.967
	Linear PFOS	2.53 ± 0.19	12.69 ± 0.37	0.998	2.59 ± 0.49	0.36 ± 0.04	0.928
	Branched PFAS	1.02 ± 0.07	15.03 ± 0.17	0.993	0.97 ± 0.09	0.23 ± 0.02	0.949
Designer biochar	PFOA	7.52 ± 0.01	3.70 ± 0.37	0.986	5.41 ± 0.26	0.37 ± 0.02	0.989
	Linear PFOS	15.36 ± 3.08	2.09 ± 0.71	0.937	7.59 ± 0.46	0.40 ± 0.01	0.987
	Branched PFAS	4.51 ± 0.11	6.49 ± 0.14	0.989	3.32 ± 0.85	0.31 ± 0.07	0.922

Abbreviation: PFAS, per‐ and polyfluoroalkyl substances.

### 3.6 Improved sorption mechanisms of PFAS by designer biochar compared to biochar

**FIGURE 6:**
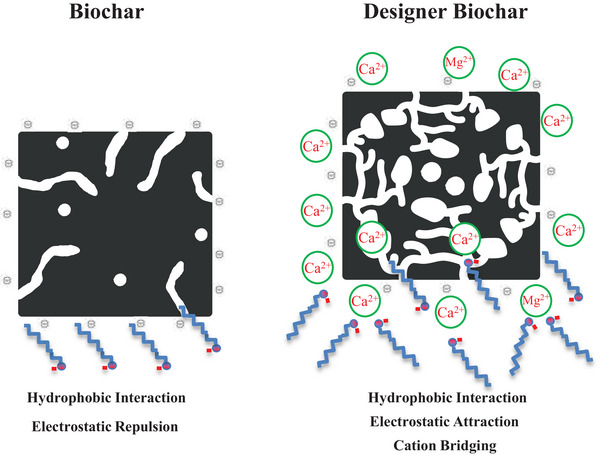
Adsorption mechanisms of per‐ and polyfluoroalkyl substances (PFAS) on biochar and designer biochar.

Hydrophobic and electrostatic interactions are the two principal sorption mechanisms governing the removal of PFAS by carbon‐based sorbents (Ao et al., 2024; Niaz et al., 2024; Rodrigo et al., 2022). Typically, an increased surface area and pore volume enhance hydrophobic adsorption by providing more active sorption sites. The physicochemical properties of unmodified and lime sludge‐incorporated designer biochars are summarized in Table S3. The designer biochars exhibit significantly higher surface areas and pore volumes than their unmodified biochars. For example, the designer biochar produced at 850°C for 5 h has a surface area of 224.5 m^2^ g^−^^1^ and a total pore volume of 0.122 cm^3^ g^−^^1^, compared to only 9.67 m^2^ g^−^^1^ and 0.004 cm^3^ g^−^^1^ for the unmodified biochar. These results indicate that lime sludge pretreatment promotes biochar activation and pore development during pyrolysis, generating more porous and accessible sorption sites (Figure 6). The improved structural characteristics of designer biochar facilitate hydrophobic interactions with PFAS molecules (Figure 6), resulting in significantly higher sorption affinity compared to the unmodified biochar (Figure 3). Similarly, Ao et al. (2024) reported that bamboo–biochar prepared by vacuum carbonization and steam activation exhibited superior performance for PFAS due to the development of a large pore volume, particularly within the mesopore range (Ao et al., 2024).

The pretreatment of biomass with lime sludge also alters the electrostatic characteristics of the designer biochar surface. The zeta potential values of the unmodified biochar and the designer biochar pyrolyzed at 850°C for 5 h were −27.3 ± 1.4 mV in pH 8.0 solutions and −17.0 ± 1.8 mV in pH 10.2 solutions, respectively. This indicates that lime sludge pretreatment not only increases the surface area of the designer biochar but also modifies its surface charge compared to unmodified biochar (Figure 6). The reduction in negative surface charge implies diminished electrostatic repulsion between the negatively charged hydrophilic heads of PFAS and the biochar surface, thereby increasing the potential of PFAS adsorption. Previous studies have demonstrated that electrostatic attraction serves as a favorable sorption mechanism for anionic PFAS species on sorbents exhibiting positive zeta potential values, such as Fe‐ or Al‐based hydroxides (Campos‐Pereira et al., 2020; Rodrigo et al., 2022).

Overall, lime sludge pretreatment resulted in a more open and accessible pore structure in the designer biochar. The incorporation of Ca^2^^+^ and Mg^2^^+^ species originating from lime sludge neutralizes surface negative charges and introduces additional cationic bridging sites (Figure 6). Consequently, PFAS adsorption onto the designer biochar occurs through three primary mechanisms, including (i) enhanced hydrophobic interactions, (ii) electrostatic attraction, and (iii) cation bridging facilitated by Ca^2^^+^ and Mg^2^^+^ species (Figure 6). The synergistic effect of these mechanisms accounts for the significantly enhanced PFAS sorption capacity of the designer biochar compared with the unmodified biochar.

## 4 CONCLUSION

This study demonstrated that lime sludge pretreatment of woody biomass prior to pyrolysis significantly enhances the sorptive performance of designer biochar for PFAS removal from water. The optimized designer biochar, produced at 850°C for 5 h with a 1:4 lime sludge‐to‐biomass ratio, achieved >99% removal of PFOA and PFOS, exhibiting faster sorption kinetics and higher capacities than the unmodified biochar. The improvement was attributed to substantial increases in surface area and pore volume, reduced surface negativity, and the incorporation of Ca^2^^+^ and Mg^2^^+^ species that promote electrostatic attraction and cation bridging with anionic PFAS. Compared with post‐modification techniques, lime sludge pretreatment provides a simple, cost‐effective, and scalable strategy for producing high‐performance sorbents. Beyond its technical efficacy, this method utilizes a drinking water treatment byproduct as an additive, contributing to improved environmental sustainability and safer sorbent production. Future studies using complex water matrices are needed to further evaluate the performance of the optimized designer biochar under dynamic, environmentally relevant conditions for PFAS removal.

## AUTHOR CONTRIBUTIONS

**Wei Zheng**: Conceptualization; funding acquisition; methodology; resources; supervision; writing—original draft; writing—review and editing. **Erin Huggett**: Data curation; formal analysis; investigation; methodology; validation. **Sophie Circenis**: Data curation; investigation; methodology. **Kalidas Mainali**: Data curation; formal analysis; investigation; methodology. **Brajendra Sharma**: Formal analysis; investigation; methodology; writing—review and editing.

## CONFLICT OF INTEREST STATEMENT

The authors declare no conflicts of interest.

## Supporting information



Supplementary Material

## References

[jeq270195-bib-0001] Afrooz, M. , Zeynali, R. , Soltan, J. , & McPhedran, K. N. (2025). A novel biochar adsorbent for treatment of perfluorooctanoic acid (PFOA) contaminated water: Exploring batch and dynamic adsorption behavior. Journal of Water Process Engineering, 69, 106586. 10.1016/j.jwpe.2024.106586

[jeq270195-bib-0002] Ao, W. Y. , Mian, M. M. , Zhang, Q. X. , Zhou, Z. M. , & Deng, S. B. (2024). Bamboo‐derived low‐cost mesoporous biochar for efficient removal of per‐ and polyfluoroalkyl substances from contaminated water. ACS ES&T Water, 4, 2711–2720. 10.1021/acsestwater.4c00211

[jeq270195-bib-0003] Boone, J. S. , Vigo, C. , Boone, T. , Byrne, C. , Ferrario, J. , Benson, R. , Donohue, J. , Simmons, J. E. , Kolpin, D. W. , Furlong, E. T. , & Glassmeyer, S. T. (2019). Per‐ and polyfluoroalkyl substances in source and treated drinking waters of the United States. Science of the Total Environment, 653, 359–369. 10.1016/j.scitotenv.2018.10.245 30412881 PMC6996027

[jeq270195-bib-0004] Čabala, R. , Nesměrák, K. , & Vlasáková, T. (2017). Dissociation constants of perfluoroalkanoic acids. Monatshefte für Chemie—Chemical Monthly, 148, 1679–1684. 10.1007/s00706-017-1970-4

[jeq270195-bib-0005] Campos‐Pereira, H. , Kleja, D. B. , Sjöstedt, C. , Ahrens, L. , Klysubun, W. , & Gustafsson, J. P. (2020). The adsorption of per‐ and polyfluoroalkyl substances (PFASs) onto ferrihydrite is governed by surface charge. Environmental Science & Technology, 54, 15722–15730. 10.1021/acs.est.0c01646 33244971 PMC7745537

[jeq270195-bib-0006] Crone, B. C. , Speth, T. F. , Wahman, D. G. , Smith, S. J. , Abulikemu, G. , Kleiner, E. J. , & Pressman, J. G. (2019). Occurrence of per‐ and polyfluoroalkyl substances (PFAS) in source water and their treatment in drinking water. Critical Reviews in Environmental Science and Technology, 49, 2359–2396. 10.1080/10643389.2019.1614848 32831535 PMC7433796

[jeq270195-bib-0007] Deng, J. , Han, J. , Hou, C. , Zhang, Y. , Fang, Y. , Du, W. , Li, M. , Yuan, Y. , Tang, C. , & Hu, X. (2023). Efficient removal of per‐ and polyfluoroalkyl substances from biochar composites: Cyclic adsorption and spent regenerant degradation. Chemosphere, 341, 140051. 10.1016/j.chemosphere.2023.140051 37660789

[jeq270195-bib-0008] Domingo, J. L. , & Nadal, M. (2019). Human exposure to per‐and polyfluoroalkyl substances (PFAS) through drinking water: A review of the recent scientific literature. Environmental Research, 177, 108648. 10.1016/j.envres.2019.108648 31421451

[jeq270195-bib-0009] Du, Z. W. , Deng, S. B. , Chen, Y. G. , Wang, B. , Huang, J. , Wang, Y. J. , & Yu, G. (2015). Removal of perfluorinated carboxylates from washing wastewater of perfluorooctanesulfonyl fluoride using activated carbons and resins. Journal of Hazardous Materials, 286, 136–143. 10.1016/j.jhazmat.2014.12.037 25585266

[jeq270195-bib-0010] Giesy, J. P. , & Kannan, K. (2001). Global distribution of perfluorooctane sulfonate in wildlife. Environmental Science & Technology, 35, 1339–1342. 10.1021/es001834k 11348064

[jeq270195-bib-0011] Hansen, M. C. , Børresen, M. H. , Schlabach, M. , & Cornelissen, G. (2010). Sorption of perfluorinated compounds from contaminated water to activated carbon. Journal of Soils and Sediments, 10, 179–185. 10.1007/s11368-009-0172-z

[jeq270195-bib-0012] Huang, P. J. , Hwangbo, M. , Chen, Z. Y. , Liu, Y. N. , Kameoka, J. , & Chu, K. H. (2018). Reusable functionalized hydrogel sorbents for removing long‐ and short‐chain perfluoroalkyl acids (PFAAs) and GenX from aqueous solution. ACS Omega, 3, 17447–17455. 10.1021/acsomega.8b02279 31458350 PMC6644158

[jeq270195-bib-0013] Inyang, M. , & Dickenson, E. R. V. (2017). The use of carbon adsorbents for the removal of perfluoroalkyl acids from potable reuse systems. Chemosphere, 184, 168–175. 10.1016/j.chemosphere.2017.05.161 28586657

[jeq270195-bib-0014] Johnson, J. K. , Hoffman, C. M. , Smith, D. A. , & Xia, Z. Y. (2019). Advanced filtration membranes for the removal of perfluoroalkyl species from water. ACS Omega, 4, 8001–8006. 10.1021/acsomega.9b00314 31459888 PMC6648049

[jeq270195-bib-0015] Katuwal, S. , Circenis, S. , Zhao, L. D. , & Zheng, W. (2023). Enhancing dissolved inorganic phosphorous capture by gypsum‐incorporated biochar: Synergic performance and mechanisms. Journal of Environmental Quality, 52, 949–959. 10.1002/jeq2.20505 37555696

[jeq270195-bib-0016] Krebsbach, S. , He, J. Z. , Adhikari, S. , Olshansky, Y. , Feyzbar, F. , Davis, L. C. , Oh, T. S. , & Wang, D. J. (2023). Mechanistic understanding of perfluorooctane sulfonate (PFOS) sorption by biochars. Chemosphere, 330, 138661. 10.1016/j.chemosphere.2023.138661 37044140

[jeq270195-bib-0017] Krebsbach, S. , He, J. Z. , Oh, T. S. , & Wang, D. J. (2023). Effects of environmental factors on the sorption of per‐ and polyfluoroalkyl substances by biochars. ACS ES&T Water, 3, 3437–3446. 10.1021/acsestwater.3c00458

[jeq270195-bib-0018] Li, S. W. , Oliva, P. , Zhang, L. , Goodrich, J. A. , McConnell, R. , Conti, D. V. , Chatzi, L. , & Aung, M. (2025). Associations between per‐and polyfluoroalkyl substances (PFAS) and county‐level cancer incidence between 2016 and 2021 and incident cancer burden attributable to PFAS in drinking water in the United States. Journal of Exposure Science & Environmental Epidemiology, 35, 425–436. 10.1038/s41370-024-00742-2 39789195 PMC12069088

[jeq270195-bib-0019] Lindstrom, A. B. , Strynar, M. J. , Delinsky, A. D. , Nakayama, S. F. , McMillan, L. , Libelo, E. L. , Neill, M. , & Thomas, L. (2011). Application of WWTP biosolids and resulting perfluorinated compound contamination of surface and well water in Decatur, Alabama, USA. Environmental Science & Technology, 45, 8015–8021. 10.1021/es1039425 21513287

[jeq270195-bib-0020] Liu, N. , Wu, C. , Lyu, G. F. , & Li, M. Y. (2021). Efficient adsorptive removal of short‐chain perfluoroalkyl acids using reed straw‐derived biochar (RESCA). Science of the Total Environment, 798, 149191. 10.1016/j.scitotenv.2021.149191 34333431

[jeq270195-bib-0021] Liu, Z. Y. , Zhang, P. , Wei, Z. X. , Xiao, F. , Liu, S. , Guo, H. , Qu, C. C. , Xiong, J. , Sun, H. W. , & Tan, W. F. (2023). Porous Fe‐doped graphitized biochar: An innovative approach for co‐removing per‐/polyfluoroalkyl substances with different chain lengths from natural waters and wastewater. Chemical Engineering Journal, 476, 146888. 10.1016/j.cej.2023.146888

[jeq270195-bib-0022] Mccleaf, P. , Englund, S. , Östlund, A. , Lindegren, K. , Wiberg, K. , & Ahrens, L. (2017). Removal efficiency of multiple poly‐ and perfluoroalkyl substances (PFASs) in drinking water using granular activated carbon (GAC) and anion exchange (AE) column tests. Water Research, 120, 77–87. 10.1016/j.watres.2017.04.057 28478297

[jeq270195-bib-0023] McGregor, R. (2018). In Situ treatment of PFAS‐impacted groundwater using colloidal activated carbon. Remediation—The Journal of Environmental Cleanup Costs, Technologies, & Techniques, 28, 33–41. 10.1002/rem.21558

[jeq270195-bib-0024] Nakayama, S. F. , Yoshikane, M. , Onoda, Y. , Nishihama, Y. , Iwai‐Shimada, M. , Takagi, M. , Kobayashi, Y. , & Isobe, T. (2019). Worldwide trends in tracing poly‐ and perfluoroalkyl substances (PFAS) in the environment. TrAC Trends in Analytical Chemistry, 121, 115410. 10.1016/j.trac.2019.02.011

[jeq270195-bib-0025] Nasrollahpour, S. , Pulicharla, R. , & Brar, S. K. (2025). Functionalized biochar for the removal of poly‐ and perfluoroalkyl substances in aqueous media. iScience, 28, 112113. 10.1016/j.isci.2025.112113 40160421 PMC11951031

[jeq270195-bib-0026] Niaz, W. , Zhang, D. H. , Ahmad, Z. , Shen, N. , Haider, W. , Ali, I. , Usman, M. , Majid, A. , Javaid, S. F. , Amjed, M. A. , & Li, X. G. (2024). Efficient removal of perfluorooctanoic acid (PFOA) and perfluorooctane sulfonic acid (PFOS) from aqueous solution using modified biochar: Preparation, performance, and mechanistic insights. Journal of Environmental Chemical Engineering, 12, 114894. 10.1016/j.jece.2024.114894

[jeq270195-bib-0027] Petrangeli Papini, M. , Senofonte, M. , Cuzzola, R. A. , Remmani, R. , Pettiti, I. , Riccardi, C. , & Simonetti, G. (2024). Adsorption technology for PFAS removal in water: Comparison between novel carbonaceous materials. Materials, 17, 4169. 10.3390/ma17174169 39274559 PMC11395723

[jeq270195-bib-0028] Rehman, A. , Park, M. , & Park, S. J. (2019). Current progress on the surface chemical modification of carbonaceous materials. Coatings, 9, 103. 10.3390/coatings9020103

[jeq270195-bib-0029] Remucal, C. K. (2019). Spatial and temporal variability of perfluoroalkyl substances in the Laurentian Great Lakes. Environmental Science: Processes & Impacts, 21, 1816–1834. 10.1039/c9em00265k 31347638

[jeq270195-bib-0030] Rodrigo, P. M. , Navarathna, C. , Pham, M. T. H. , McClain, S. J. , Stokes, S. , Zhang, X. F. , Perez, F. , Gunatilake, S. R. , Karunanayake, A. G. , Anderson, R. , Thirumalai, R. , Mohan, D. , Pittman, C. U. , & Mlsna, T. E. (2022). Batch and fixed bed sorption of low to moderate concentrations of aqueous per‐ and poly‐fluoroalkyl substances (PFAS) on Douglas fir biochar and its Fe_3_O_4_ hybrids. Chemosphere, 308, 136155. 10.1016/j.chemosphere.2022.136155 36099986

[jeq270195-bib-0031] Ross, I. , McDonough, J. , Miles, J. , Storch, P. , Kochunarayanan, P. T. , Kalve, E. , Hurst, J. , Dasgupta, S. S. , & Burdick, J. (2018). A review of emerging technologies for remediation of PFASs. Remediation—The Journal of Environmental Cleanup Costs, Technologies, & Techniques, 28, 101–126. 10.1002/rem.21553

[jeq270195-bib-0032] Schulz, K. , Silva, M. R. , & Klaper, R. (2020). Distribution and effects of branched versus linear isomers of PFOA, PFOS, and PFHxS: A review of recent literature. Science of the Total Environment, 733, 139186. 10.1016/j.scitotenv.2020.139186 32474294

[jeq270195-bib-0033] Tokranov, A. K. , Ransom, K. M. , Bexfield, L. M. , Lindsey, B. D. , Watson, E. , Dupuy, D. I. , Stackelberg, P. E. , Fram, M. S. , Voss, S. A. , Kingsbury, J. A. , Jurgens, B. C. , Smalling, K. L. , & Bradley, P. M. (2024). Predictions of groundwater PFAS occurrence at drinking water supply depths in the United States. Science, 386, 748–755. 10.1126/science.ado6638 39446898

[jeq270195-bib-0034] Wang, Y. , Niu, J. , Li, Y. , Zheng, T. , Xu, Y. , & Liu, Y. (2015). Performance and mechanisms for removal of perfluorooctanoate (PFOA) from aqueous solution by activated carbon fiber. RSC Advances, 5, 86927–86933. 10.1039/C5RA15853B

[jeq270195-bib-0035] Wang, Z. Y. , Alinezhad, A. , Nason, S. , Xiao, F. , & Pignatello, J. J. (2023). Enhancement of per‐ and polyfluoroalkyl substances removal from water by pyrogenic carbons: Tailoring carbon surface chemistry and pore properties. Water Research, 229, 119467. 10.1016/j.watres.2022.119467

[jeq270195-bib-0036] Wang, Z. Y. , Alinezhad, A. , Sun, R. Z. , Xiao, F. , & Pignatello, J. J. (2023). Pre‐ and postapplication thermal treatment strategies for sorption enhancement and reactivation of biochars for removal of per‐ and polyfluoroalkyl substances from water. ACS ES&T Engineering, 3, 193–200. 10.1021/acsestengg.2c00271

[jeq270195-bib-0037] Wilder, L. , Worley, R. , & Breysse, P. (2017). Community exposures to per‐ and polyfluoroalkyl substances in drinking water: A national issue. Journal of Environmental Health, 80, 38–41.

[jeq270195-bib-0038] Xiao, X. , Ulrich, B. A. , Chen, B. L. , & Higgins, C. P. (2017). Sorption of poly‐ and perfluoroalkyl substances (PFASs) relevant to aqueous film‐forming foam (AFFF)‐impacted groundwater by biochars and activated carbon. Environmental Science & Technology, 51, 6342–6351. 10.1021/acs.est.7b00970 28582977

[jeq270195-bib-0039] Yang, S. M. , Katuwal, S. , Zheng, W. , Sharma, B. , & Cooke, R. (2021). Capture and recover dissolved phosphorous from aqueous solutions by a designer biochar: Mechanism and performance insights. Chemosphere, 274, 129717. 10.1016/j.chemosphere.2021.129717 33529948

[jeq270195-bib-0040] Yao, Y. , Gao, B. , Chen, J. J. , & Yang, L. Y. (2013). Engineered biochar reclaiming phosphate from aqueous solutions: Mechanisms and potential application as a slow‐release fertilizer. Environmental Science & Technology, 47, 8700–8708. 10.1021/es4012977 23848524

[jeq270195-bib-0041] Yu, H. , Chen, H. , Zhang, P. , Yao, Y. M. , Zhao, L. C. , Zhu, L. Y. , & Sun, H. W. (2023). In situ self‐sacrificial synthesis of polypyrrole/biochar composites for efficiently removing short‐ and long‐chain perfluoroalkyl acid from contaminated water. Journal of Environmental Management, 344, 118745. 10.1016/j.jenvman.2023.118745 37562255

[jeq270195-bib-0042] Zhang, D. Q. , He, Q. C. , Wang, M. , Zhang, W. L. , & Liang, Y. N. (2019). Sorption of perfluoroalkylated substances (PFASs) onto granular activated carbon and biochar. Environmental Technology, 42(12), 1798–1809. 10.1080/09593330.2019.1680744 31625466

[jeq270195-bib-0043] Zhao, L. , Zheng, W. , Mašek, O. , Chen, X. , Gu, B. , Sharma, B. K. , & Cao, X. (2017). Roles of phosphoric acid in biochar formation: Synchronously improving carbon retention and sorption capacity. Journal of Environmental Quality, 46, 393–401. 10.2134/jeq2016.09.0344 28380545

