# Supplementary material for: Designing biochars for improved sorptive removal of per‐ and polyfluoroalkyl substances
Source: J Environ Qual. 2026 May 13;55:e70195. doi: 10.1002/jeq2.70195 (PMC13172648; doi:10.1002/jeq2.70195)
Supplement: Supplementary file 1 — Supplementary Material [file JEQ2-55-0-s001.pdf]

Supplementary Material

Designing Biochars for Improved Sorptive Removal of Per- and Polyfluoroalkyl Substances

Wei Zheng [1,*]; Erin Huggett [1], Sophie Circenis [1], Kalidas Mainali [2], and Brajendra Sharma [2]

[1] Illinois Sustainable Technology Center, University of Illinois at Urbana-Champaign, Champaign, IL 61820, USA.

[2] U.S. Department of Agriculture, Agricultural Research Service, Eastern Regional Research Center, Sustainable Biofuels and Co-Products Research Unit, PA 19038, USA

* Correspondence: weizheng@illinois.edu

Total 11 pages, including 6 tables and 2 figures.

Text S1: **Sorption kinetics and isotherms models**

Three kinetic models, pseudo-first order, pseudo-second order, and Elovich, were applied to fit experimental data in order to compare the sorption kinetics of PFOA and PFOS on unmodified biochar and designer biochar.

Pseudo-first-order model:

$$\frac{dQ_t}{dt} = k_1(Q_e - Q_t) \tag{1}$$

Pseudo-second-order model:

$$\frac{dQ_t}{dt} = k_2(Q_e - Q_t)^2 \tag{2}$$

Elovich model:

$$\frac{dQ_t}{dt} = \alpha \exp(-\beta Q_t) \tag{3}$$

where $Q_e$and Q_t(mg g^{-1}) represent the amounts of PFAS sorbed on the sorbents at equilibrium and at time t(h), respectively; k_1(min^{-1}) and k_2(g mg^{-1} min^{-1}) are the rate constants of the pseudo-first-order and pseudo-second-order models; α(mg g^{-1} min^{-1}) is the initial sorption rate; and β(g mg^{-1}) is the desorption constant.

Two nonlinear isotherm models were used to describe the equilibrium sorption behavior of PFAS on unmodified biochar and designer biochar.

Langmuir model:

$$Q_e = \frac{Q_{\max} K_L C_e}{1 + K_L C_e} \tag{4}$$

Freundlich model:

$$Q_e = K_f C_e^{1/n} \tag{5}$$

where Q_e (mg g^{-1}) is the sorption capacity at the equilibrium concentration C_e (mg L^{-1}); $Q_{\max}$ (mg g^{-1}) is the maximum sorption capacity; K_L (L mg^{-1}) and K_f (mg$^{(1-n)}$ L^{n} g^{-1}) are the Langmuir and Freundlich constants, respectively; and n is the Freundlich heterogeneity factor.

Table S1. Optimized MS parameters, retention times, instrumentation limit of detection (LOD), and limit of quantification (LOQ) used for targeted PFAS Quantification

Target Analyte List	Acronym	MRM ions (m/z)	Q1 Pre Bias (V)	Collision Energy (V)	Q3 Pre Bias (V)	Retention Time (min)	LOD (ng/ml)	LOQ (ng/ml)
Perfluorooctanoic Acid	PFOA	413>369	29.0	10.0	17.0	6.50	0.08	0.26
Perfluoro-n-[1,2,3,4-13C4]octanoic acid	$^{13}C_4$-PFOA	421>376	29.0	10.0	17.0	6.50		
Perfluorooctanesulfonic Acid	PFOS LIN	499>80	10.0	50.0	29.0	8.23	0.17	0.56
	PFOS ISO	499>80	10.0	50.0	29.0	8.13	0.19	0.63
Perfluoro-n-[1,2,3,4-13C4]octanesulfonate	$^{13}C_4$-PFOS	507>80	10.0	50.0	29.0	8.23		

Table S2. Effects of lime sludge (LS) pretreatment ratios with biomass on PFAS sorption capacities (mg PFAS per g biochar, mg/g)

**Sorbent**	**PFOA**	**Linear PFOS**	**Branched PFOS**
Unmodified Biochar	0.119 ± 0.084 [a]	0.270 ± 0.059 [a]	0.082 ± 0.031 [a]
Designer biochar (1:20 LS: biomass)	0.476 ± 0.034 [b]	0.830 ± 0.007 [c]	0.337 ± 0.014 [b]
Designer biochar (1:4 LS: biomass)	1.239 ± 0.034 [c]	0.834 ± 0.011 [c]	0.398 ± 0.016 [c]
Designer biochar (1:1 LS: biomass)	1.236 ± 0.012 [c]	0.852 ± 0.017 [c]	0.398 ± 0.020 [c]
Activated carbon	1.230 ± 0.004 [c]	0.852 ± 0.002 [c]	0.397 ± 0.031 [c]

Sorption capacities between two sorbents followed by different letters are significantly different ($p < 0.05$)

Table S3: Selected physicochemical properties of commercial activated carbon, unmodified biochars, and designer biochars prepared under different conditions from selected feedstocks.

	Pyrolysis conditions	Carbon (%)	Hydrogen (%)	Nitrogen (%)	Oxygen (%)	Ash (%)	Surface area (m^2/g)	Pore volume (cm^3/g)
Biochar	650 °C, 5hr, No LS	86.37	1.55	0.58	8.67	2.83	4.79	0.003
Designer Biochar	650 °C, 5hr, 1:4 LS: Biomass	46.03	0.98	0.51	8.35	44.13	104.9	0.053
Biochar	850 °C, 5hr, No LS	91.81	0.67	0.72	5.28	1.52	9.67	0.004
Designer Biochar	850 °C, 5hr, 1:4 LS: Biomass	42.73	1.04	0.44	2.50	53.29	224.5	0.122
Biochar	900 °C, 5hr, No LS	87.47	0.58	0.82	8.21	2.92	12.0	0.007
Designer Biochar	900 °C, 5hr, 1:4 LS: Biomass	44.01	0.79	0.62	7.09	47.49	150.9	0.080
Biochar	1000 °C, 5hr, No LS	89.69	0.5	1.01	5.67	3.13	14.5	0.010
Designer Biochar	1000 °C, 5hr, 1:4 LS: Biomass	50.11	0.54	0.49	6.45	42.41	124.7	0.079
Biochar	850 °C, 2hr, No LS	89.21	0.58	0.77	6.99	2.45	9.18	0.011
Designer Biochar	850 °C, 2hr, 1:4 LS: Biomass	43.59	0.85	0.48	11.52	43.56	169.3	0.080
Commercial Activated Carbon		89.69	0.50	1.01	5.80	3.00	912.4	0.528

LS: Lime Sludge

Table S4. Best-fit parameters of sorption kinetics models for biochar and designer biochar applied to PFOA.

Model	Designer biochar			Biochar		
	Parameter 1	Parameter 2	R^2	Parameter 1	Parameter 2	R^2
**Pseudo-First Order**	k_1=0.197 mg g^{-1}	Q_e = 2.669 mg g^{-1}	0.898	k_1=0.095 mg g^{-1}	Q_e = 1.537 mg g^{-1}	0.880
**Pseudo-Second Order**	k_2=0.112 g mg^{-1} min^{-1}	Q_e = 2.837 mg g^{-1}	0.961	k_2= 0.087 g mg^{-1} min^{-1}	Q_e = 1.755 mg g^{-1}	0.940
**Elovich**	β = 0.098g mg^{-1}	α=3.883 x 10^4 mg g^{-1} min^{-1}	0.987	β = 0.117 g mg^{-1}	α=2.788 x 10^2 mg g^{-1} min^{-1}	0.986

Table S5. Best-fit parameters of sorption kinetics models for biochar and designer biochar applied to linear PFOS.

Model	Designer biochar			Biochar		
	Parameter 1	Parameter 2	R^2	Parameter 1	Parameter 2	R^2
**Pseudo-First Order**	k_1=0.123 mg g^{-1}	Q_e = 3.302 mg g^{-1}	0.992	k_1=0.028 mg g^{-1}	Q_e = 2.580 mg g^{-1}	0.936
**Pseudo-Second Order**	k_2=0.121 g mg^{-1} min^{-1}	Q_e = 3.332 mg g^{-1}	0.979	k_2= 0.017 g mg^{-1} min^{-1}	Q_e = 2.746 mg g^{-1}	0.967
**Elovich**	β = 6.077 g mg^{-1}	α= 3.04 x 10^5 mg g^{-1} min^{-1}	0.959	β = 3.716g mg^{-1}	α= 8.912 mg g^{-1} min^{-1}	0.953

Table S6. Best-fit parameters of sorption kinetics models for biochar and designer biochar applied to branched PFOS.

Model	Designer biochar			Biochar		
	Parameter 1	Parameter 2	R^2	Parameter 1	Parameter 2	R^2
**Pseudo-First Order**	k_1=0.124 mg g^{-1}	Q_e = 1.345 mg g^{-1}	0.957	k_1=0.014 mg g^{-1}	Q_e = 0.934 mg g^{-1}	0.938
**Pseudo-Second Order**	k_2=0.162 mg g^{-1}	Q_e = 1.400 mg g^{-1}	0.969	k_2=0.022 mg g^{-1}	Q_e = 1.018 mg g^{-1}	0.974
**Elovich**	β = 10.171g mg^{-1}	α= 3.76 x 10^2 mg g^{-1} min^{-1}	0.939	β = 5.966 g mg^{-1}	α= 0.067 mg g^{-1} min^{-1}	0.951

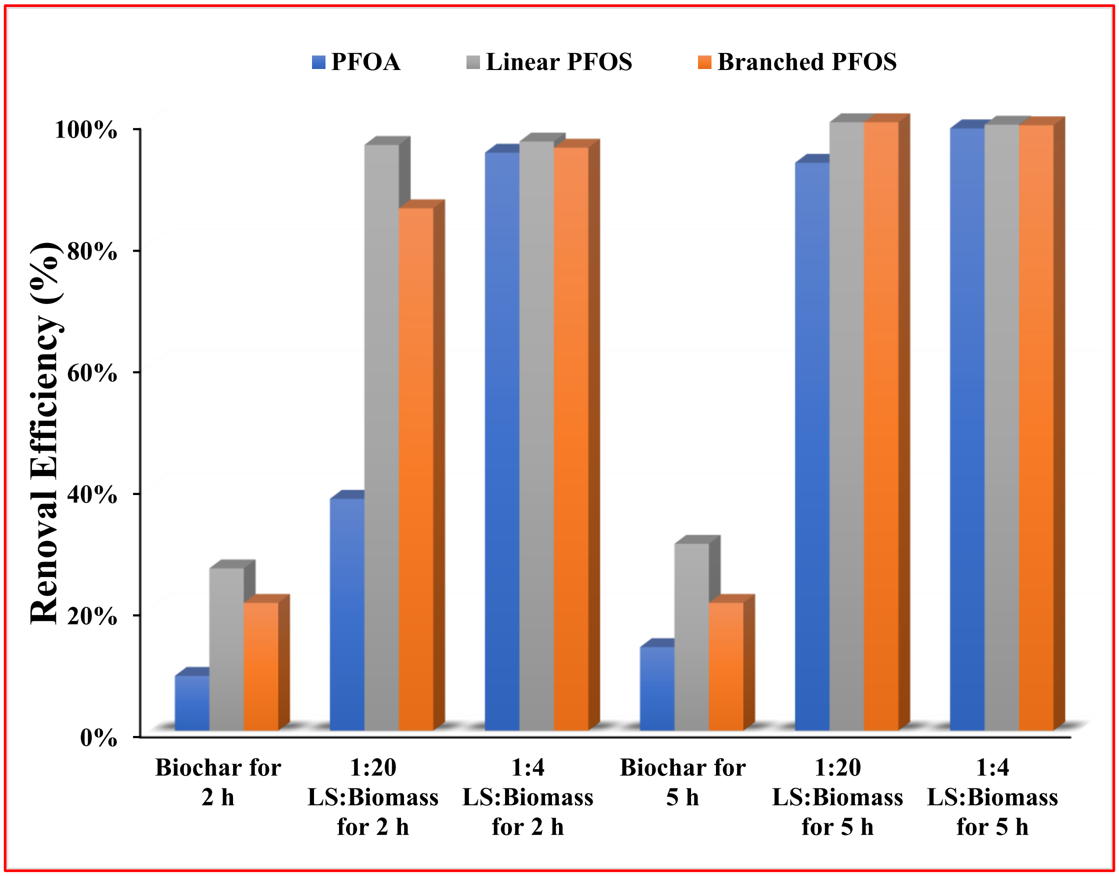

Figure S1. Effect of pyrolysis duration on PFOA and PFOS removal by the raw biochars and the designer biochars produced from 850 °C pyrolysis temperature

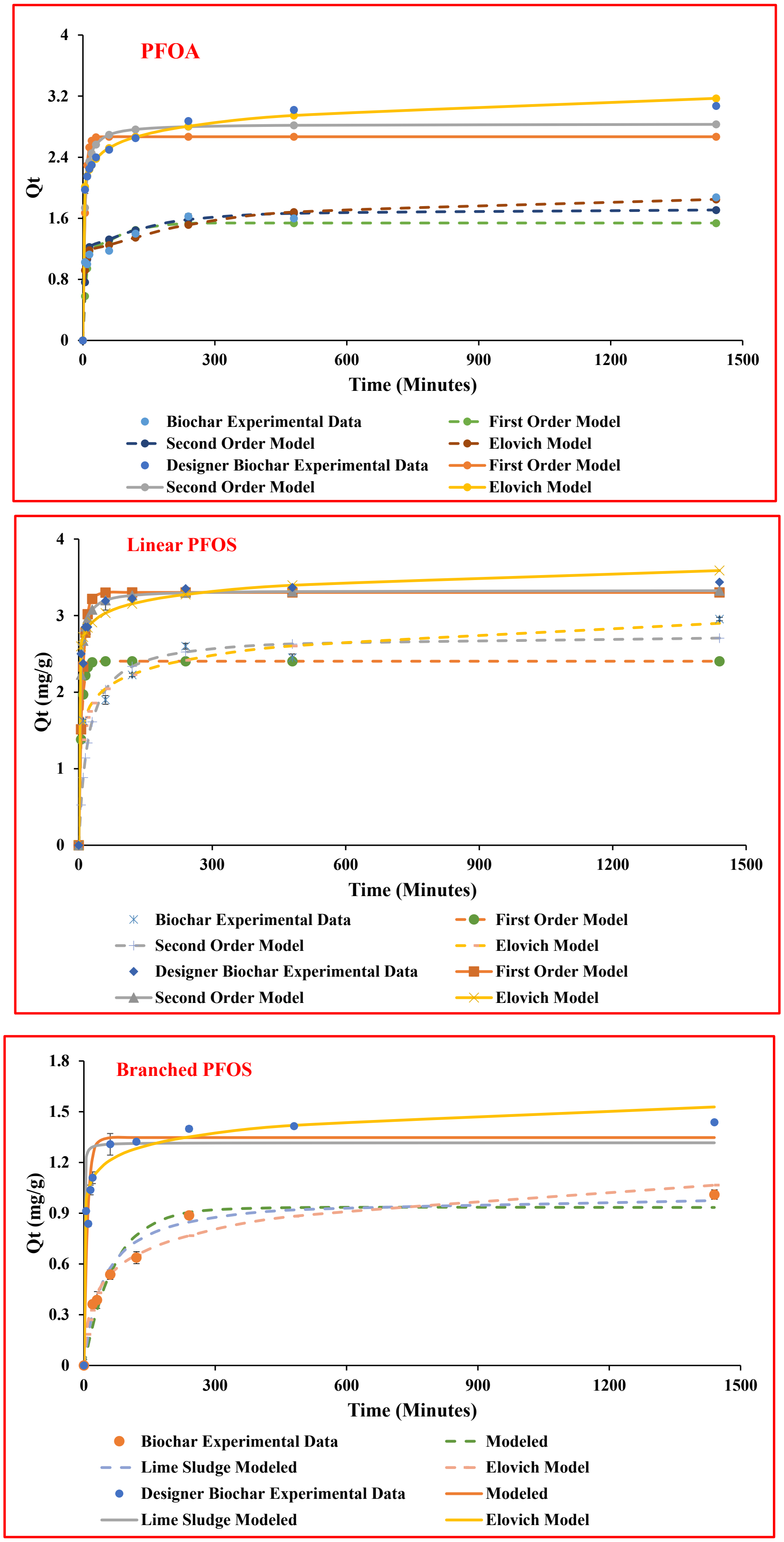

Figure S2. Sorption kinetics and models of PFOA, Linear PFOS, and Branched PFOS by biochar and designer biochar.
